# Transcriptome Profiles of Human Lung Epithelial Cells A549 Interacting with *Aspergillus fumigatus* by RNA-Seq

**DOI:** 10.1371/journal.pone.0135720

**Published:** 2015-08-14

**Authors:** Fangyan Chen, Changjian Zhang, Xiaodong Jia, Shuo Wang, Jing Wang, Yong Chen, Jingya Zhao, Shuguang Tian, Xuelin Han, Li Han

**Affiliations:** Department for Hospital Infection Control & Research, Institute of Disease Control & Prevention of PLA, Academy of Military Medical Sciences, Beijing, China; Hans-Knoell-Institute (HKI), GERMANY

## Abstract

Lung epithelial cells constitute the first defense line of host against the inhaled *Aspergillus fumigatus*; however, the transcriptional response of human alveolar type II epithelial cells was still unclear. Here we used RNA-Seq technology to assess the transcriptome profiles of A549 cells following direct interaction with conidia of *A*. *fumigatus*. The total number of identified genes was 19118. Compared with uninfected A549 cells, 459 genes were differentially expressed in cells co-incubated with conidia for 8 h, including 302 up-regulated genes and 157 down-regulated genes. GO and KEGG pathway enrichment analysis showed that most of the up-regulated genes were related to immune response, chemotaxis and inflammatory response and enriched in cytokine-cytokine receptor interaction, JAK-STAT and MAPK signaling pathways. The down-regulated genes were mainly enriched for terms associated with development, hemopoiesis and ion transport. Among them, EGR4 and HIST1H4J gene had the maximum of fold change in up-regulated and down-regulated genes, respectively. Fourteen up-regulated genes and three down-regulated genes were further validated and significant increase on expression of IL-6, IL-8 and TNF-α in A549 cells were confirmed by qRT-PCR during the interaction of A549 cells with *A*. *fumigatus*. Besides, western blot showed that expression of two proteins (ARC, EGR1) significantly increased in A549 cells during interaction with *A*. *fumigatus* conidia for 8h. Interference of endogenous expression of ARC or EGR1 protein in A549 cells reduced the internalization of *A*. *fumigatus*. These results provided important insights into dynamic changes of gene expression in lung epithelial cells, especially its strong immunological response against *A*. *fumigatus* infection.

## Introduction

*Aspergillus fumigatus* is an important opportunistic fungal pathogen, widely distributed in nature including soil, decaying vegetation, compost heaps, and in most indoor environments [1–3]. The diameter of conidiophores of *A*. *fumigatus* is about 2∼5μm, easily floating in the air and allowing them to reach the innermost areas of the lung, including the alveoli [1, 4]. It is likely for all humans to inhale at least several hundred conidia each day [5]. Depending on the immune status of the host, *Aspergillus* species can cause a spectrum of diseases in humans which range from local hypersensitivity reactions to often fatal systemic mycoses [6]. *A*. *fumigatus* is responsible for approximately 90% of human *Aspergillus* infection [1, 7–8].

Lung epithelial cells have been demonstrated to be not only so much the “innocent bystanders” as a purely physical barrier but also the important member of the first defense line of the host innate immune system like alveolar macrophage, neutrophil, etc [9]. They play an important role in releasing inflammatory factors, presenting signal to lymphocytes and even directly killing microbes [10]. Because of lack of a better system almost all the involvement of lung epithelial cells in innate immunity response against infection was evidenced in lung carcinoma A549 cell line which is type II-like lung epithelial cells covering only less than 5% of the alveolar surface [9]. We have also demonstrated that *A*. *fumigatus* conidia can internalize into A549 cells and human bronchial epithelial cell line 16HBE14o, which is closely related to phospholipase D activation and actin cytoskeleton rearrangement in host cells [11–12]. The internalization of *A*. *fumigatus* conidia also induces innate immune response including release of inflammatory factors and respiratory burst [10]. Many studies have shown that *A*. *fumigatus* conidia could survive and disseminate within these normally non-phagocytic host cells upon evasion of host defense by phagocytes [13–15]. This may be an important reason for the spread of *A*. *fumigatus* infection, further causing invasive aspergillosis, but the specific regulation mechanism is still unclear.

Recently some studies used microarray technology to investigate the gene expression profiles of host cells interacting with *A*. *fumigatus* [4, 16–17]. The microarray technology can simultaneously detect thousands of transcription, and has become the major tool to study gene expression for a time. However, microarray technology is based on hybrid method, the bias and noise hinder its widely use for high-throughput technique [18–19]. With the development of high-throughput new generation sequencing technologies, the transcriptome sequencing (RNA-Seq) technology gradually became the new mainstream approach for analysis of the transcriptome. It can complete transcription fragment sequencing in the manner of unbiasedness and single-base resolution in the whole genome, which is called a revolutionary tool in the field of the transcriptome research [20]. A number of studies have applied whole genome transcriptional profiling to study interactions between mammalian hosts and microbes [21–25]. To our knowledge, there is no report using RNA-Seq technology to study the alteration of the transcriptome of human alveolar type II epithelial cell line A549 interacting with *A*. *fumigatus*.

In the present study, we used RNA-Seq technology to assess the cell-specific transcriptional response of cultured human alveolar epithelial cells (A549) following interaction with *A*. *fumigatus*, which would contribute to better understand the process at the molecular level and provide theoretical foundation for diagnosis and treatment of varieties of pulmonary fungal diseases.

## Materials and Methods

### *A*. *fumigatus* strain, cell line and culture conditions

*A*. *fumigatus* wild type strain B5233 was generously provided by Dr. KJ.Kwon-Chung (National Institute of Health, Bethesda, Maryland) [26]. Except where indicated, the *A*. *fumigatus* strain was propagated on Sabouraud dextrose agar slants (10 g/L peptone, 10 g/L glucose, 15 g/L agar, pH 6.0) for 5–8 days at 37°C. *A*. *fumigatus* conidia were harvested and prepared in the same way as described in a previous study [11]. After 5–8 days of cultivation, *A*. *fumigatus* conidia were dislodged from agar plates with gentle washing. They were then re-suspended in sterile phosphate-buffered saline supplemented with 0.1% Tween 20 (PBST). The conidia were passed through 8 layers of sterile gauze in order to remove hyphal fragments. At this point, they were enumerated on a hemacytometer and the resting conidia were washed twice and stored at 4°C to be used within 48 h. The type II human pneumocyte cell line A549 was obtained from the ATCC and cultured in Dulbecco Modified Eagle Medium (DMEM) supplemented with a 10% fetal calf serum (GIBCO, Germany), 100 mg/L streptomycin, 16 mg/L penicillin at 37°C in 5% CO_2_.

### Exposure of epithelial cells to *A*. *fumigatus* conidia

A549 cells were grown at a density of 2 x 10^6^ cells/150 mm dish, and cultured in serum-free DMEM for 16 to 18 h before treatment. Then cells were incubated in serum-free DMEM for 8 h with *A*. *fumigatus* wild type strain B5233 resting conidia (multiplicity of infection between conidia and A549 cells was ten, MOI = 10) at 37°C in 5% CO_2_. The uninfected A549 cells were cultured under the same medium conditions.

### RNA preparation and RNA-Seq analysis

Upon the completion of incubation, the culture supernatant was removed and cells were washed three times in sterile phosphate-buffered saline (PBS) to remove any conidia that did not bind/attach to cells. Total RNA was prepared from infected and uninfected A549 cells with TRIzol (Sigma Chemical Co., St. Louis, MO) in the dish (1ml TRIzol per 10 cm^2^), respectively. The samples were stored at -80°C prior to RNA extraction. RNA extraction and RNA-Seq analysis were performed by BGI Tech company (Shenzhen, China). Briefly, after the total RNA extraction and DNase I treatment, the mRNA was fragmented into short fragments. Then cDNA was synthesized using the mRNA fragments as templates. After agarose gel electrophoresis, the suitable fragments were selected for the PCR amplification as templates. During the QC steps, Agilent 2100 Bioanaylzer and ABI StepOnePlus Real-Time PCR System were used in quantification and qualification of the sample library. At last, the library could be sequenced using Illumina HiSeq 2000.

Primary sequencing data that produced by Illumina HiSeq 2000, is called as raw reads. Before doing any further analysis, quality control was required in order to detect whether the data is qualified. In addition, filtering of raw data was needed to decrease data noise. Several softwares updated were used to perform these tasks. After filtering, the remaining reads were called "clean reads" and used for downstream bioinformatics analysis. Clean reads were mapped to a reference sequences using *SOAPaligner/SOAP2*. No more than 5 mismatches were allowed in the alignment. The gene expression level was calculated by using *RPKM* method (Reads per kilobase transcriptome per million mapped reads), and *p* value was calculated according to the Poisson distribution. We used *p* value < 0.05 and the fold change ≥ 1.5 as the threshold to judge the significance of gene expression difference. Further, we used DAVID online tool (http://david.abcc.ncifcrf.gov) to perform Gene Ontology (GO) enrichment analysis and KEGG Pathway enrichment analysis [27–28]. Our dataset were deposited in SRA repository, and the accession number is SRP051309.

### qRT-PCR assay

Total RNA was isolated from 1×10^6^ A549 cells by RNApure Tissue Kit (with DNase I) (CW BIOtech, CW0560, China) following the manufacturer’s protocols. RNA was used to synthesize cDNA using Transcriptor First Strand cDNA Synthesis Kit (Roche, Cat.No.04896866001) according to the manufacturer’s protocols. For RT-PCR, A549 cells were cultured on 35-mm cell-culture plates for 24 h in Dulbecco’s modified Eagle’s medium (DMEM); 1.2 ml of Buffer RL (CW BIOtech, CW0560, China) was added to each plate, and RNA was isolated according to the protocol supplied by the manufacturer. To ensure the quality of the isolated RNA, the RNA samples were analyzed by gel electrophoresis. Total RNA (2μg in a total volume of 20μl) was denatured at 65°C for 10 min and used for reverse transcription as described by the standard protocol (Roche). cDNA (2μl in a total volume of 20μl) was used as the template for qPCR. Glyceraldehyde phosphate dehydrogenase (GAPDH) was the reference gene. These genes specific primers were designed using Primer5 software (Table 1). And the specificity of the primers were tested by NCBI Primer-Blast (http://www.ncbi.nlm.nih.gov/tools/primer-blast/). qRT-PCR was performed using SYBR *Premix Ex Taq* (Tli RNaseH Plus) (TaKaRa, cat.#RR420A, Japan) according to the manufacturer’s protocols and reaction was performed by Bio-Rad iQ5 Detection System (version:2.0.148.60623). The thermal cycling conditions were: initial denaturation at 95°C for 3 min, followed by 40 cycles of 95°C for 10 s and 55°C for 30 s, then followed by 95°C for 1 min and 55°C for 1 min. We used the “delta delta Ct” method for analyzing the qRT-PCR data. The fold change was derived by calculating the ratio between the experimental group and control.

**Table 1:** Primers used in the study.

Gene Symbol	Primer Sequence (From 5' to 3')
EGR1	F: TCGGACATGACAGCAACCT
	R: CATCTGACCTAAGAGGAACCC
CCR4	F: AGGACCTTATCTGTGCGTGAA
	R: GCCTGGCTCAACATGCTACTA
ARC	F:GGTTCATCGTTCTGCCTTGT
	R:GAGGATTGGTTATGGCTTATGT
PTGS2	F: CTCAGCCATACAGCAAATCC
	R: TCCGGGTACAATCGCACT
COLEC12	F: GATGGGATCACATGAGCAAA
	R: TGGGTAATGACGGATGGTAAA
FOS	F: AGGACCTTATCTGTGCGTGAA
	R:CAACATGCTACTAACTACCAGCTCT
EGR4	F: AGCAAGAGATGGGTTTATG
	R: AGGAGTTGGAAGAAGAGC
LST1	F: GCATTGCTGAGAACAAACCC
	R: GATCAATTTGAACGGAGGC
DUSP2	F:TACCTCATGCAGAGTCGCC
	R:CAGCACCTGGGTCTCAAACT
CREB5	F: GGGACCACAGGTTCACATAA
	R: TTGGGAGGAAGAAGTAGGATAG
CCL20	F: GCCCAAGAACAGAAAGAACC
	R: CAAGTCCAGTGAGGCACAAA
ABL2	F:CCTCCTCGTCATCTGTTGTTC
	R:CTTGTTCTCCACCTGTTTCTTC
JUN	F: CTGCGTCTTAGGCTTCTCCA
	R: GCTCGCCCAAGTTCAACAA
ROCK1P1	F: GAAAATGGCTGTCAGTCGTG
	R:GATGGCCGTTCCACTTGAAAGTGAA
HOXA9	F:CCGAGCAAAAGACGAGTGA
	R:GATGTGGCCTGAGGTTTAGAG
CALCB	F: TCTTTCGGAGCCATCCTG
	R: GTCCTCGGAGACCACAACA
LY6D	F: TGGGGATTCCACACCTCTCT
	R: GACCTGGTCCCAGACTTTCG
IL6	F: CATTCCTTCTTCTGGTCAG
	R: TAGTGTCCTAACGCTCATA
IL8	F: TGCCAGCTGTGTTGGTAGTG
	R: TGACTGTGGAGTTTTGGCTGT
TNF-α	F: CCCAGGGACCTCTCTCTAATCA
	R: GCTACAGGCTTGTCACTCGG
GAPDH	F: GCCCTCAACGACCACTTTGT
	R: TGGTGGTCCAGGGGTCTTAC

### Western Blotting

Western blotting analyses were performed on cell protein of uninfected- and B5233 conidia-infected A549 cells. 20μg of total protein was separated on SDS-PAGE gels. Proteins were transferred to PVDF membranes. The membranes were blocked with 5% (w/v) nonfat milk in TBS tween buffer (TBST, 0.1%, v/v) for 1h and then incubated with primary antibodies either EGR1(dilution 1:200) or ARC(dilution 1:200) at 4°C overnight or at room temperature(RT) 2-3h. β-actin (dilution 1:1000) was used as a loading control. All the primary antibodies were purchased from Santa Cruz. After three subsequent washes in TBST at RT, the membranes were incubated with secondary HRP-antibodies at RT for 1h and washed again. Chemiluminescence detection was performed by ECL (Pierce, Rockford, USA).

### RNA interference

For ARC and ERG1 interference, antisense RNA oligomers were designed and chemically synthesized by Sigma-Aldrich. The siRNA ID for ARC and ERG1 were as follows: ARC, SASI_Hs02_00347261; EGR1, SASI_Hs01_00232227. The siRNA universal negative control (Sigma) was used. For transfection, the cells were plated in 35 mm dishes and transfected with 5 μL lipofectamin 2000 Reagent (Invitrogen) in 100 μL Opti-MEM containing 100 pmol of ARC or ERG1 siRNA.

### Analysis of *A*. *fumigatus* internalization

The nystatin protection assay to determine the *A*. *fumigatus* internalization into A549 cells were performed as previously described [11–12]. For the nystatin protection assay, A549 cells were seeded at 1 × 10^4^ cells/well in 96-well plates (Corning) and grown for 16 h. The cells were incubated with *A*. *fumigatus* resting conidia at the indicated MOI. Internalization was performed by incubation at 37°C for the indicated times in 5% CO2. Subsequently, the wells were washed 3 times with PBST (PBS containing 0.1% Tween-20) and incubated with nystatin (20 μg/ml) in DMEM for 4 h at 37°C. The monolayers were washed twice with PBST and lysed by incubating in 0.25% Triton X-100 for 15 min. The released conidia were diluted and plated onto SDA agar (3 replicate plates/ well) and incubated at 37°C for 24 h. The colonies were counted to determine the total intracellular conidia. The internalization capacity is expressed as a percentage of the initial inoculum.

### Statistical analysis

Data are presented as mean ± SE of three independent experiments performed in triplicate. A *t*-test was used to compare the difference between groups, and values of *P* < 0.05 were considered statistically significant, unless otherwise specified. In Gene Ontology and DAVID analysis, the statistical data were generated by the software.

## Results

### 1. Differential gene expression in A549 cells exposed to *A*. *fumigatus* conidia

To better elucidate the response of lung epithelial cells against *A*. *fumigatus* conidia infection at the molecular level, we used RNA-Seq analysis to determine changes in gene expression in A549 cells infected with *A*. *fumigatus* conidia. In this analysis total number of identified genes was 19118. Among them, 459 genes were differentially expressed (*p* value < 0.05, fold change 1.5 or greater). Compared to uninfected A549 cells, there were 302 up-regulated genes and 157 down-regulated genes in A549 cells infected with *A*. *fumigatus* conidia (S1 and S2 Tables). Moreover, when the threshold value of fold change was taken as 2 or greater, the number of differential expressed genes was still 318 (206 up-regulated; 112 down-regulated).

**Fig 1:**
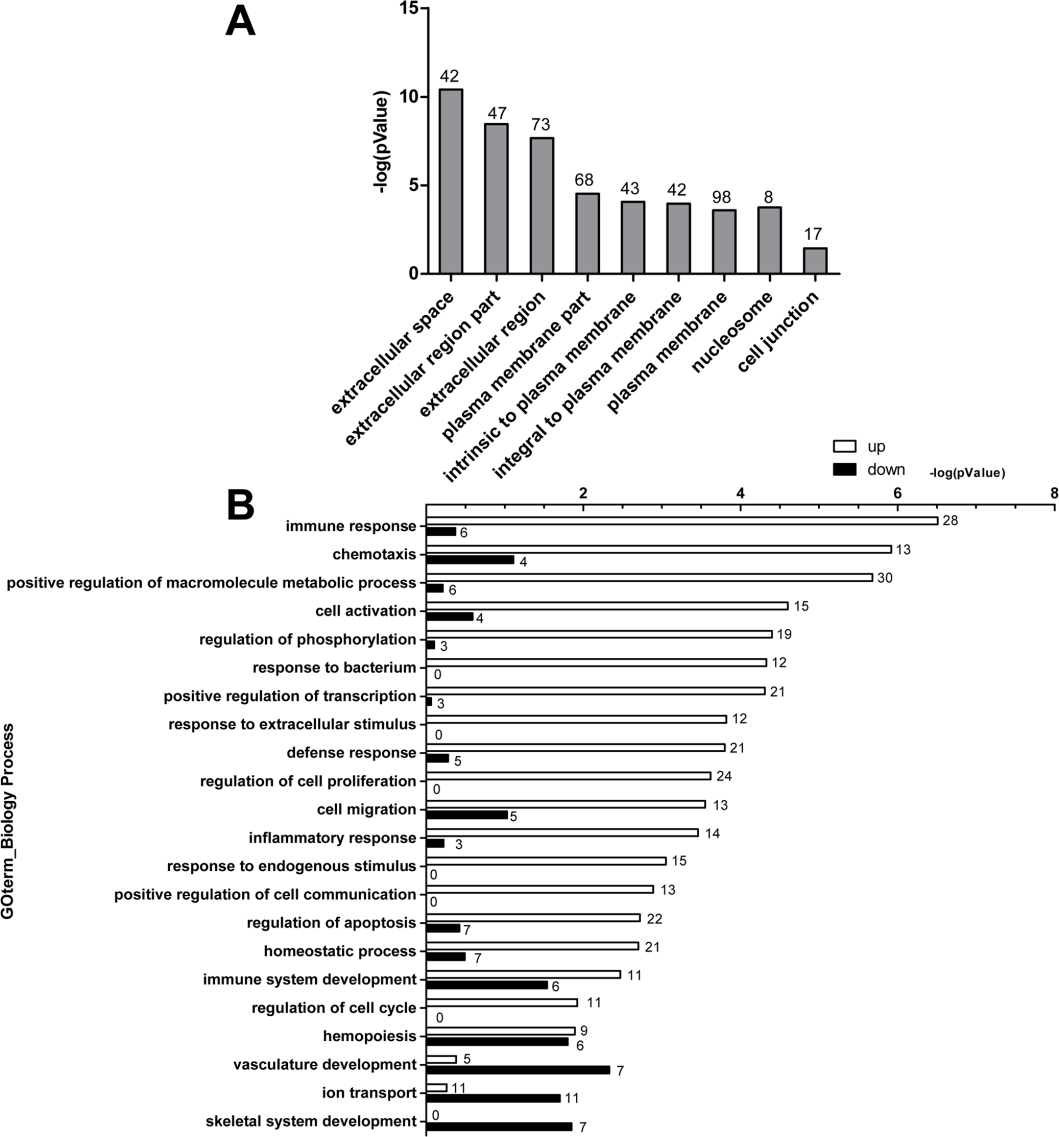
Bioinformatics analysis of differential expressed genes. Bioinformatics analysis was provided by DAVID Bioinformatics Resources 6.7 (http://david.abcc.ncifcrf.gov/). The differential expressed genes were determined with *p* value ≤ 0.05 and fold change 1.5 or greater. **(A)** Result of subcellular localization analysis, the number above the pillar in graph is the number of genes encoding proteins with indicated subcellular localization. **(B)** Result of GO biology process enrichment analysis of up/down-regulated genes and the number above the pillar in graph is the number of genes of participation in the biology process.

To determine the major biological themes in this dataset, the Gene Ontology (GO) enrichment analysis and KEGG pathway enrichment analysis were provided by DAVID Bioinformatics Resources 6.7 (http://david.abcc.ncifcrf.gov). GO enrichment analysis showed that the differentially expressed genes were mainly located in extracellular region, plasma membrane (Fig 1A). The up-regulated genes were mainly enriched for terms associated with immune response, chemotaxis, positive regulation of macromolecule metabolic process, cell activation, regulation of phosphorylation, response to bacterium, positive regulation of transcription, response to extracellular or endogenous stimulus and defense response, inflammatory response (Fig 1B). The up-regulated gene with the maximum of fold change (12.01, *p* value = 1.81E-153) was EGR4 (early growth response 4) (S1 Table). EGR4 protein is a transcriptional regulator, belongs to EGRs family. The other three members of EGRS family were also obviously up-regulated, including EGR1 (fold change = 8.69, *p* value = 0), EGR2 (fold change = 7.29, *p* value = 0) and EGR3 (fold change = 8.15, *p* value = 7.54E-212). Interestingly, we found that five members of DUSPs (dual-specificity phosphatases) family were also up-regulated, including DUSP-1 (fold change = 1.53, *p* value = 0), DUSP-2 (fold change = 3.12, *p* value = 4.18E-05), DUSP-5 (fold change = 1.94, *p* value = 0), DUSP-6 (fold change = 2.14, *p* value = 8.58E-183), and DUSP-8 (fold change = 4.68, *p* value = 5.4E-194) (S1 Table). The down-regulated genes were mainly enriched for terms associated with vasculature development, skeletal system development, hemopoiesis, ion transport and immune system development (Fig 1B). The down-regulated gene with the maximum of fold change (8.10, *p* value = 1.21E-3) was HIST1H4J (S2 Table). HIST1H4J protein is one of the core components of nucleosome. KEGG pathway enrichment analysis showed that differential expressed genes were mainly enriched for terms related to cytokine-cytokine receptor interaction, JAK-STAT signaling pathway, MAPK signaling pathway and chemokine signaling pathway, most of which encode chemokine, inflammatory cytokine, transcriptional factor and transcriptional regulators (Table 2).

**Table 2:** List of differential expressed genes enriched in KEGG Pathway.

GOID	KEGG_Pathway	*P* value	Differential gene expression in A549 cells following exposure to *A*. *fumigatus* conidia
470038830	Cytokine-cytokine receptor interaction	1.40E-11	CXCL2,CXCL3,CCL20,CCL28,CXCR4,CXCR6,CCR4,IL1A,IL1B,IL6,IL8,IL11,IL20,IL24,CNTF,CSF2,CSF3,TSLPR,TNF,LTA,LTB,INHBA,TNFSF13B,EDAR
470038866	Jak-STAT signaling pathway	4.00E-05	IL6,IL11,IL20,IL24,Sprouty1,Sprouty2,Sprouty4,SOCS1,CNTF,CSF2,CSF3,CRLF2
470038827	MAPK signaling pathway	9.90E-05	DDIT3,CACNA1B,DUSP1,DUSP2,DUSP5,DUSP6,DUSP8,IL1A,IL1B,c-JUN,NR4A1,PLA2G10,PTPRR,FOS,TNF
470038867	Hematopoietic cell lineage	4.90E-04	FCGR1C,CSF2.CSF3,IL1A,IL1B,IL6,IL11,TNF
470038916	Systemic lupus erythematosus	1.10E-03	FCGR1C, Hist1h2ae,Hist1h2ah, Hist1h3i, Hist1h4b, HIST2H2BF, HIST3H2BB,TNF
470038863	NOD-like receptor signaling pathway	3.40E-03	CXCL2,IL1B,IL6,IL8,TNF,TNFAIP3
470038831	Chemokine signaling pathway	3.40E-03	CCL20,CCL28,CCR4,CXCL2,CXCL3,CXCR4,CXCR6,GNG13,IL8,ROCK1
470038874	Intestinal immune network for IgA production	8.30E-03	CCL28,CXCR4,ITGB7,IL6,TNFSF13B,APRIL,CXCL12,IL4
470038895	Prion diseases	1.90E-02	EGR1,IL1A,IL1B,IL6
470038862	Toll-like receptor signaling pathway	2.50E-02	IL1B,IL6,IL8,c-JUN,TNF,FOS
470038829	Calcium signaling pathway	2.50E-02	Htr2b,HTR7,Cacna1b,Chrm5,grpr,GNA15,NOS1, SLC8A2
470038918	Graft-versus-host disease	2.60E-02	IL1A,ILB,IL6,TNF
470038888	Type I diabetes mellitus	3.10E-02	IL1A,IL1B,lta,TNF

In previous studies we found that *A*. *fumigatus* conidia could induce obvious actin cytoskeleton rearrangement of A549 cells [11–12]. Interestingly, some up-regulated genes in this RNA-Seq analysis play important role in cytoskeleton remodeling, regulation of actin polymerization and morphological changes. Function of these proteins was provided by UniProt (http://www.uniprot.org/). For example, protein coded by ABL2 (gene name, v-abl Abelson murine leukemia viral oncogene homolog 2; fold change = 1.58; *p* value = 0) is a non-receptor tyrosine-protein kinase that plays an ABL1-overlapping role in key processes linked to cell growth and survival such as cytoskeleton remodeling in response to extracellular stimuli, cell motility and adhesion and receptor endocytosis. This kinase can coordinate actin remodeling through tyrosine phosphorylation of proteins controlling cytoskeleton dynamics like MYH10 (involved in cell movement); CTTN (involved in cell signaling); or TUBA1 and TUBB (microtubule subunits). The ABL2 kinase also can bind directly F-actin and regulate actin cytoskeletal structure through its F-actin-bundling activity. ARC (gene name, activity-regulated cytoskeleton-associated protein; fold change = 3.42; *p* value = 1.59E-66) codes a protein, which functions as a component of the Arp2/3 complex which is involved in regulation of actin polymerization and together with an activating nucleation-promoting factor (NPF) mediates the formation of branched actin networks. LST1 (gene name, leukocyte specific transcript 1; fold change = 7.55; *p* value = 8.91E-3) codes a protein that induces morphological changes including production of filopodia and microspikes when overexpressed in a variety of cell types. Taken together, these results suggested that A549 cells have a significant response to infection of *A*. *fumigatus*, including immune response and actin cytoskeleton rearrangement.

### 2. Validation by qRT-PCR

**Fig 2:**
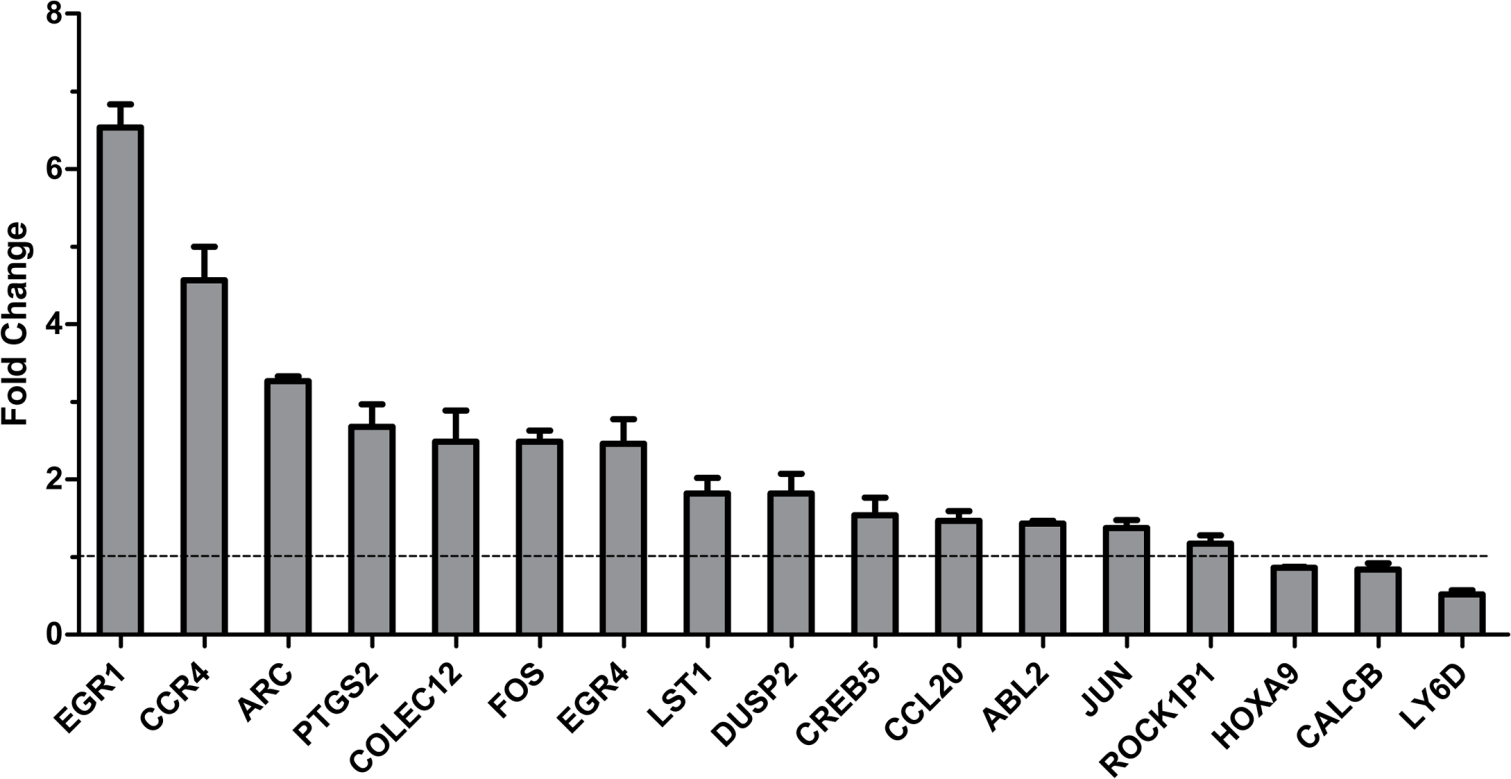
Validation of RNA-Seq analysis results by qRT-PCR. A549 cells were infected with or without the living resting conidia of wild type *A*. *fumigatus* B5233 at an MOI of 10 for 8 h at 37°C. The cDNA samples of the cells were prepared for qRT-PCR assay as the same as performed in RNA-Seq assay. Fourteen up-regulated genes and three down-regulated genes shown in RNA-Seq analysis data were examined by qRT-PCR, respectively. Height of each bar represents fold change of gene in infected condition relative to cells alone control. The fold change greater than 1 means that the gene was up-regulated, in contrast, the fold change less than 1 means that the gene was down-regulated. Data are represented as the mean ± SE (n = 3–4).

In order to validate the results from RNA-Seq analysis, according to biology process classification, seventeen differential expressed genes, including fourteen up-regulated genes and three down-regulated genes, were chosen for further validation by qRT-PCR (Table 3). Housekeeping gene GAPDH was used as a reference gene. As shown in Fig 2, relative to the uninfected cells, the fold change of fourteen genes was more than 1, including EGR1, CCR4, ARC, PTGS2, COLEC12, FOS, EGR4, LST1, DUSP2, CREB5, CCL20, ABL2, JUN and ROCK1P1. They displayed remarkably up-regulated in A549 cells infected with *A*. *fumigatus* conidia; while three genes, HOXA9, CALCB and LY6D showed a significant down-regulation (the fold change of them was less than 1) (Fig 2). These results were consistent with results from RNA-Seq analysis, despite some differences of multiple is not precisely consistent. The expression of CALCB in A549 cells with infection by conidia had also downtrend. These results confirmed the expression pattern observed in the RNA-Seq analysis.

**Table 3:** Differential expressed genes were further validated by qRT-PCR.

Gene Symbol	Gene Name	Biology Process	Fold Change	*P* value
EGR4	early growth response 4	regulation of cell proliferation; positive regulation of transcription; positive regulation of macromolecule metabolic process;	12.01	1.81E-153
EGR1	early growth response 1	positive regulation of transcription; cell activation;	8.69	0
CCR4	chemokine (C-C motif) receptor 4	inflammatory response; defense response; response to bacterium; chemotaxis; immune response; homeostatic process; cell migration;	7.84	1.80E-06
LST1	leukocyte specific transcript 1	immune response; regulation of cell proliferation;	7.55	9.81E-03
FOS	v-fos FBJ murine osteosarcoma viral oncogene homolog	inflammatory response; defense response; response to extracellular/endogenous stimulus; positive regulation of transcription; positive regulation of macromolecule metabolic process;	7.53	0
COLEC12	collectin sub-family member 12	defense response; immune response;	5.37	1.49E-02
ARC	activity-regulated cytoskeleton-associated protein	Regionalization; endocytosis; pattern specification process; endoderm development;	3.42	1.59E-66
CCL20	chemokine (C-C motif) ligand 20	inflammatory response; defense response; response to bacterium; chemotaxis; immune response	3.22	9.02E-31
DUSP2	dual specificity phosphatase 2	regulation of phosphorylation;	3.12	4.18E-05
PTGS2	prostaglandin-endoperoxide synthase 2	response to extracellular/endogenous stimulus; response to bacterium; regulation of apoptosis; positive regulation of cell communication; regulation of cell proliferation;	3.07	0
CREB5	cAMP responsive element binding protein 5	positive regulation of transcription; positive regulation of macromolecule metabolic process;	3.02	2.13E-27
ROCK1P1	Rho-associated, coiled-coil containing protein kinase 1 pseudogene 1	regulation of apoptosis; cell migration;	2.56	2.81E-3
JUN	jun oncogene	response to extracellular stimulus; positive regulation of transcription; response to bacterium; regulation of phosphorylation; positive regulation of macromolecule metabolic process; homeostatic process; regulation of apoptosis; regulation of cell proliferation;	1.98	0
ABL2	v-abl Abelson murine leukemia viral oncogene homolog 2 (arg, Abelson-related gene)	phosphate metabolic process; cytoskeleton organization; actin filament organization; cell adhesion; phosphorylation;	1.58	0
CALCB	calcitonin-related polypeptide beta	cellular calcium ion homeostasis; signal transduction;	-7.15	6.26E-05
LY6D	lymphocyte antigen 6 complex, locus D	cell adhesion; lymphocyte differentiation; response to stilbenoid;	-4.77	2.57E-05
HOXA9	homeobox A9	skeletal system development; immune system development; hemopoiesis	-4.01	1.08E-09

### 3. Internalization of *A*. *fumigatus* into epithelial cells and genes related to cytoskeleton rearrangement

**Fig 3:**
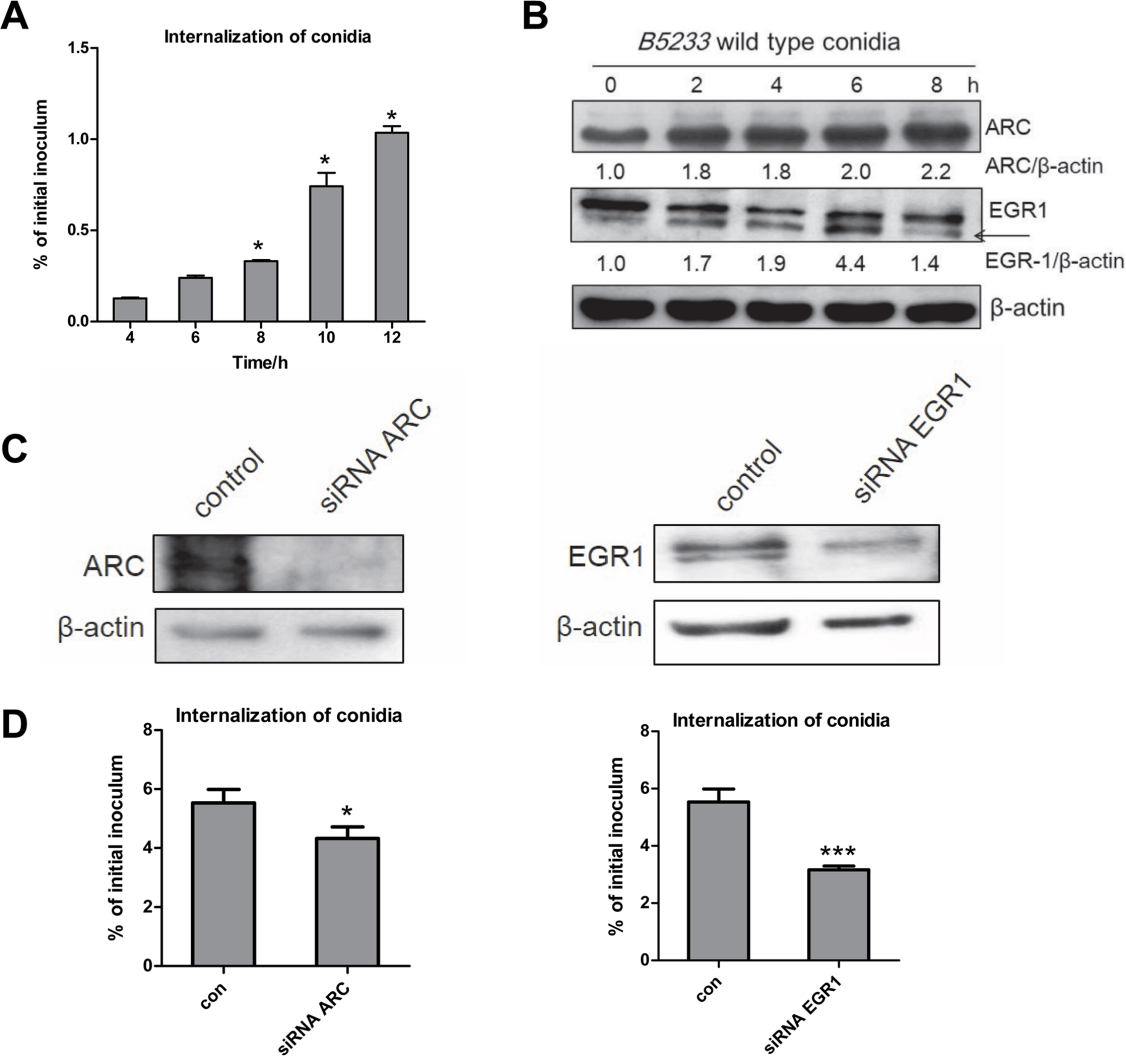
Change of expression actin-cytoskeleton related-protein affected internalization *A*. *fumigatus*. A549 cells were infected with living resting conidia of wild type *A*. *fumigatus* B5233 at an MOI of 10 at 37°C in 5% CO_2_ for the indicated time, respectively. **(A)** *A*. *fumigatus* internalization was analyzed by the nystatin protection assay. Differences of the internalization of *A*. *fumigatus* conidia between the 4 h time point and the other time points were compared. **(B)** The expression of EGR1 and ARC protein were detected by western blot. **(C, D)** A549 cells were transfected with non-specific small interfering RNAs (siRNAs) (Control), EGR1-specific siRNAs or ARC-specific siRNAs. After 48 h, cells were infected with the conidia of *A*. *fumigatus* B5233 at an MOI of 10. *A*. *fumigatus* internalization was analyzed by the nystatin protection assay. Data are represented as the mean ± SE (n = 3–4). *P< 0.05. The western blots represent EGR1 and ARC expression in A549 cell lysates.

It is known that *A*. *fumigatus* is able to internalize into lung epithelial cells accompained with actin cytoskeleton rearrangement; hereby we investigated further the transcriptomic response of genes and the internalization of *A*. *fumigatus* in A549 cells. Indeed, according to the results of Gene Ontology (GO) and KEGG analysis, a major of differential expressed genes in A549 cells after *A*. *fumigatus* infection referred to the expression of some proteins involved in cytoskeleton rearrangement. We used nystatin protection assay to determine the percentage of *A*. *fumigatus* internalization into A549 cells, meanwhile detect the expression of two cytoskeletion rearrangement-related proteins, ARC and EGR1 by western blot. It was found that *A*. *fumigatus* conidia internalized into A549 cells increasingly in a time-dependent manner (Fig 3A). Compared with 4 h, the internalization of conidia into A549 cells significantly increased when infection time was 8 h or longer. Expectedly, the expression of ARC and EGR1 protein increased obviously during *A*. *fumigatus* conidia infection (Fig 3B).

In addition, to testify whether endogenous ERG1 or ARC is involved in *A*. *fumigatus* internalization, we reduced the expression of endogenous ERG1 and ARC with small interfering RNAs (siRNAs). As illustrated in Fig 3C and 3D, the internalization of conidia into A549 cells expressing siRNAs targeting either ARC or ERG1 was diminished by approximately 20% and 40% respectively as compared with cells transfected with control siRNA. These results indicated that A549 cells have significant response in actin cytoskeleton rearrangement during *A*. *fumigatus* internalization.

## Discussion

In the present study, it is the first time to use RNA-Seq technology to analyze the transcriptome response of A549 cells infected with *A*. *fumigatus* conidia for 8 h. After adding conidia into cell culture plate, these conidia germinate in culture medium. It is difficult to distinguish the transcriptomic response to internalized conidia from the response to the growing, non-internalized germlings, In addtion, during the germination and growth, *A*. *fumigatus* can also produce some effective factors to stimulate host cells, leading to effective biological responses, like many proteases and toxins [12]. Therefore, our results revealed a global response of lung epithelial cells to *A*. *fumigatus*, including direct and indirect interaction between *A*. *fumigatus* and host cells.

Compared with uninfected cells, 459 differentially expressed genes stated a clearly-association with immunological response in A549 cells by GO enrichment analysis (Fig 1B). We also proved that expression of three inflammatory cytokines, IL6, IL-8 and TNF-α in A549 cells augmented clearly during infection by *A*. *fumigatus* (S1 Fig). All these data was in line with previous findings that the genes involved in intracellular signaling pathways and the secretion of inflammatory cytokines were activated after 8 h of exposure to *A*. *fumigatus* conidia [8]. Similarly, the results from Oosthuizen JL, *et al*. indicated that the innate immune response is one of the prevalent themes in the differentially expressed genes of lung epithelial cells after 6 h of exposure to *A*. *fumigatus* conidia; nevertheless, the number of differentially expressed genes at 6h of exposure is less than at 8 h of exposure to *A*. *fumigatus* conidia [8, 17]. More, it had been reported that interaction with growing germ tube, rather than with conidia of *A*. *fumigatus*, led to stronger activation of immune responses in A549 cells [29]. The longer the incubation time was, the more exposure of fungal antigens on the surface of conidia which got swollen and germinated during the incubation, leading to higher immune response in macrophages and dendritic cells [30]. Definitely we verified that *A*. *fumigatus* conidia get germination and tube growth at 8 h obviously more than 6 h incubation with A549 cells (data not shown). However, previous study also reported that the inflammatory response of lung epithelial cells decreased at 24 h post *A*. *fumigatus* infection [14, 31]. Taken together, it can be deduced that germination of *A*. *fumigatus* could induce more cytokines release and stronger immune response of lung epithelial cells against infection of *A*. *fumigatus*; when the conidia get inside epithelial cells or develop into mycelium, the response of epithelial cells get weaker, which helps *A*. *fumigatu*s escape the immune attack and survive. Further verification is necessary in the future study.

We previously reported that infection of *A*. *fumigatus* conidia could induce obvious actin cytoskeleton rearrangement in A549 cells [11–12]. Consistent with this, the present RNA-Seq data showed some genes associated with cytoskeleton rearrangement were up-regulated in A549 cells responsing to *A*. *fumigatus conidia*, such as ABL2, ARC and LST1. Further, this up-regulation of both ABL2 and ARC was confirmed at mRNA level by qRT-PCR and ARC protein was confirmed by western-blot in A549 cells (Figs 2 and 3B). The protein coded by the gene plays important roles in cytoskeleton rearrangement. ARC is a critical component of the Arp2/3 complex, which is proven as a key modulator on actin polymerization and cytoskeleton rearrangement. In addition, the internalization of conidia into A549 cells expressing siRNAs targeting ARC was diminished by approximately 30% as compared with cells transfected with control siRNA (Fig 3C and 3D). These results further certified that actin cytoskeleton rearrangement and its modulation is a key regulation mechanism of conidial internalization into lung epithelial cells.

EGRs are involved in modulating the immune response by means of the induction of differentiation of lymphocyte precursors, activation of T and B cells. With yeast two-hybrid screening a previous study has demonstrated that EGR4 and EGR3 are able to interact with the specific nuclear mediator NF-κB and control transcription of genes encoding inflammatory cytokines in T cells [32]. EGR1 as well as EGR4 functionally cooperate with nuclear factors of activated T cells (NFAT) proteins and induce expression of inflammatory cytokines [33]. In Toxoplasma gondii-infected host cells, EGR1 and EGR2 genes were the most rapidly up-regulated [34]. Thus, it can be deduced that the remarkable increase of inflammatory cytokines IL-6, IL-8, and TNF-α mRNA level during *A*. *fumigatus* conidia internalization into A549 cells might be related to the function of EGRs. Interestingly, we found further that the expression of EGR1, one member of EGRs family was rapidly up-regulated following infection by *A*. *fumigatus* conidia into A549 cells (Fig 3B). And the internalization of conidia into A549 cells expressing siRNAs targeting ERG1 was diminished by approximately 50% as compared with cells transfected with control siRNA (Fig 3C and 3D). Thereby, the roles of EGRs in lung epithelial cells against *A*. *fumigatus* infection are worthy of further research.

Another interesting gene family is DUSP, which currently contains 25 members and encodes dual-specificity phosphatases that dephosphorylate both tyrosine and serine/threonine residues of their substrates [35–36]. A selected group of DUSP enzymes display overexpression or hyperactivity that is associated with human disease, especially human cancer, making feasible targeted therapy approaches based on their inhibition. In the past decade, DUSPs were shown to play important roles in regulating immune cell functions *in vitro* and *in vivo* [35, 37–46]. For example, DUSP1 was involved in regulating the production of inflammatory cytokines. DUSP2 positively regulated autoimmune responses in an arthritis animal model. However, the role of DUSPs in the interaction of lung epithelial cells with *A*. *fumigatus* is unclear. In our transcriptome data, 5 members of DUSPs family were up-regulated, DUSP1, 2, 5, 6, and 8 (S1 Table). This would draw great interest to investigate the possible involvement of DUSPs in the response of lung epithelial cells to *A*. *fumigatus* in the future.

Taken together, our study provided important insights in dynamic transcriptome profile in lung epithelial cells against *A*. *fumigatus* infection. Further investigation about immune regulation mechanism in lung epithelial cells will contribute to understand the characteristics of *A*. *fumigatus* pathogenesis, and might provide some potential targets for diagnosis and treatment of pulmonary fungal diseases.

## Supporting Information

S1 Fig Alteration of host inflammatory cytokines expression in A549 cells infected by *A*. *fumigatus*. The mRNA level of inflammatory cytokines (IL-6, IL-8, TNF-α) in A549 cells was analyzed by qRT-PCR. Differences of mRNA level of inflammatory cytokines in A549 cells between the 0 h time point (uninfected A549 cells) and the other time points were compared. Data are represented as the mean ± SE (n = 3–4). *P < 0.05.
(EPS)

S1 Table Up-regulated genes in A549 cells infected with *A*. *fumigatus* conidia. 459 genes were differentially expressed, with *p* value < 0.05, fold change 1.5 or greater. The fold change for each gene is expressed as the ratio of expression between the two populations, infected A549 cells with *A*. *fumigatus* conidia and uninfected A549 cells. Compared with uninfected A549 cells, there were 302 up-regulated genes in A549 cells infected with *A*. *fumigatus* conidia. The genes were sorted by fold change.
(DOCX)

S2 Table Down-regulated genes in A549 cells infected with *A*. *fumigatus* conidia. 459 genes were differentially expressed, with *p* value < 0.05, fold change 1.5 or greater. The fold change for each gene was expressed as the ratio of expression between the two populations, infected A549 cells with *A*. *fumigatus* conidia and uninfected A549 cells. Compared with uninfected A549 cells, there were 157 down-regulated genes in A549 cells infected with *A*. *fumigatus* conidia. The genes were sorted by fold change.
(DOCX)
