# Supplementary material for: Transcriptome Profiles of Human Lung Epithelial Cells A549 Interacting with Aspergillus fumigatus by RNA-Seq
Source: PLoS One. 2015 Aug 14;10(8):e0135720. doi: 10.1371/journal.pone.0135720 (PMC4537115; doi:10.1371/journal.pone.0135720)
Supplement: S1 Table — 459 genes were differentially expressed, with p value < 0.05, fold change 1.5 or greater. The fold change for each gene is expressed as the ratio of expression between the two populations, infected A549 cells with A. fumigatus conidia and uninfected A549 cells. Compared with uninfected A549 cells, there were 302 up-regulated genes in A549 cells infected with A. fumigatus conidia. The genes were sorted by fold change. (DOCX) [file pone.0135720.s002.docx]

**Table S1: Up-regulated genes in A549 cells infected with *A. fumigatus* conidia.** 459 genes were differentially expressed, with *p* value < 0.05, fold change 1.5 or greater. The fold change for each gene is expressed as the ratio of expression between the two populations, infected A549 cells with *A. fumigatus* conidia and uninfected A549 cells. Compared with uninfected A549 cells, there were 302 up-regulated genes in A549 cells infected with *A. fumigatus* conidia. The genes were sorted by fold change.

| **GeneID** | **Gene Symbol** | **Fold Change** | **P-value** |
| --- | --- | --- | --- |
| 1961 | EGR4 | 12.00999131 | 1.81E-153 |
| 1437 | CSF2 | 10.50638292 | 1.13E-18 |
| 149647 | FAM71A | 10.26328197 | 3.30E-45 |
| 387763 | C11orf96 | 10.23282776 | 4.60E-26 |
| 387264 | KRTAP5-1 | 9.406539284 | 1.68E-10 |
| 3012 | HIST1H2AE | 8.840762751 | 0.000144102 |
| 3010 | HIST1H1T | 8.800405014 | 1.42E-05 |
| 1958 | EGR1 | 8.69383787 | 0 |
| 11009 | IL24 | 8.269224141 | 4.71E-10 |
| 100286922 | LOC100286922 | 8.196364136 | 2.37E-05 |
| 1960 | EGR3 | 8.150080716 | 7.54E-212 |
| 100192204 | PPIAP30 | 8.105332756 | 0.00317788 |
| 2354 | FOSB | 8.066839663 | 0 |
| 338651 | LOC338651 | 7.854281515 | 1.04E-08 |
| 1233 | CCR4 | 7.84395167 | 1.80E-06 |
| 55891 | LENEP | 7.742148241 | 0.0089117 |
| 6035 | RNASE1 | 7.692246643 | 0.001897694 |
| 259293 | TAS2R30 | 7.658386009 | 0.00146646 |
| 80740 | LY6G6C | 7.646642676 | 0.001897694 |
| 100874241 | STARD13-AS | 7.564696593 | 0.019312 |
| 7940 | LST1 | 7.545477409 | 0.0089117 |
| 2353 | FOS | 7.53147588 | 0 |
| 1959 | EGR2 | 7.287674751 | 0 |
| 6554 | SLC10A1 | 7.268131052 | 0.000186477 |
| 26184 | OR1F2P | 7.188337283 | 0.0089117 |
| 7288 | TULP2 | 7.0482336 | 0.000312274 |
| 284344 | LOC284344 | 7.015554031 | 0.000522934 |
| 100130075 | LOC100130075 | 7.011192704 | 0.0115323 |
| 347 | APOD | 6.96741027 | 0.0089117 |
| 4049 | LTA | 6.869830794 | 0.00317788 |
| 256957 | C17orf66 | 6.821338017 | 0.000522934 |
| 146512 | FLJ30679 | 6.717085382 | 0.000875708 |
| 5673 | PSG5 | 6.629182819 | 0.00411238 |
| 145624 | PAR1 | 6.61876903 | 0.000312274 |
| 152206 | CCDC13 | 6.583089838 | 8.61E-05 |
| 10663 | CXCR6 | 6.579352586 | 0.001897694 |
| 165140 | OXER1 | 6.566421775 | 0.00317788 |
| 100129722 | LOC100129722 | 6.537653295 | 0.00532168 |
| 126 | ADH1C | 6.432455598 | 0.01492354 |
| 282763 | OR51B5 | 6.388076939 | 0.019312 |
| 441204 | LOC441204 | 6.371816088 | 0.0115323 |
| 56120 | PCDHGB8P | 6.32364548 | 0.00532168 |
| 375686 | SPATC1 | 6.322024992 | 0.00411238 |
| 1146 | CHRNG | 6.300614098 | 0.00317788 |
| 2769 | GNA15 | 6.211828998 | 0.00532168 |
| 7124 | TNF | 6.178463123 | 0.019312 |
| 81492 | RSPH6A | 6.116902145 | 0.00317788 |
| 3363 | HTR7 | 6.0426318 | 0.000522934 |
| 124221 | PRSS30P | 6.032433693 | 0.00146646 |
| 3231 | HOXD1 | 6.021036598 | 0.01492354 |
| 140691 | TRIM69 | 5.99773988 | 0.019312 |
| 100533183 | ZNF664-FAM101A | 5.966094325 | 0.0068866 |
| 653641 | GOLGA6C | 5.940828785 | 1.09E-05 |
| 3053 | SERPIND1 | 5.930967075 | 0.0115323 |
| 4284 | MIP | 5.921092567 | 0.00532168 |
| 100996455 | LOC100996455 | 5.920428903 | 0.0089117 |
| 3083 | HGFAC | 5.904913607 | 0.019312 |
| 222537 | HS3ST5 | 5.780641759 | 0.0068866 |
| 729177 | LOC729177 | 5.725424435 | 0.019312 |
| 100302650 | BRE-AS1 | 5.724272229 | 0 |
| 3249 | HPN | 5.675660805 | 0.019312 |
| 4888 | NPY6R | 5.657742092 | 0.0068866 |
| 1259 | CNGA1 | 5.644477163 | 0.0089117 |
| 165545 | DQX1 | 5.608537459 | 0.01492354 |
| 339834 | CCDC36 | 5.584458691 | 0.0089117 |
| 3792 | KEL | 5.574797183 | 0.019312 |
| 286059 | LOC286059 | 5.533717288 | 0.019312 |
| 467 | ATF3 | 5.526401863 | 0 |
| 3662 | IRF4 | 5.394911395 | 0.000404102 |
| 646982 | LINC00598 | 5.378301151 | 0.00411238 |
| 81035 | COLEC12 | 5.366524639 | 0.01492354 |
| 8013 | NR4A3 | 5.304971511 | 0 |
| 64150 | DIO3OS | 5.304264248 | 0.0115323 |
| 57091 | CASS4 | 5.263494642 | 0.019312 |
| 3164 | NR4A1 | 5.124045065 | 0 |
| 3787 | KCNS1 | 5.116268689 | 0.00532168 |
| 285335 | SLC9C1 | 4.883134014 | 0.019312 |
| 56155 | TEX14 | 4.773360149 | 1.34E-09 |
| 26059 | ERC2 | 4.68626779 | 0.00532168 |
| 1850 | DUSP8 | 4.678526064 | 5.40E-194 |
| 92162 | TMEM88 | 4.38238456 | 2.61E-83 |
| 3576 | IL8 | 4.218965524 | 0 |
| 80117 | ARL14 | 4.201267448 | 4.23E-50 |
| 121006 | FAM186A | 4.153567021 | 0.01492354 |
| 2210 | FCGR1B | 4.131263668 | 1.58E-08 |
| 4973 | OLR1 | 4.079691388 | 2.02E-40 |
| 3552 | IL1A | 4.0543803 | 2.52E-20 |
| 100151683 | RNU4ATAC | 4.015561051 | 1.48E-05 |
| 9248 | GPR50 | 3.975668324 | 5.34E-10 |
| 1440 | CSF3 | 3.971166932 | 1.70E-14 |
| 10659 | CELF2 | 3.956144048 | 0.0089117 |
| 10252 | SPRY1 | 3.814863701 | 1.69E-28 |
| 57526 | PCDH19 | 3.803730839 | 0.0115323 |
| 285051 | C2orf61 | 3.709934768 | 3.91E-08 |
| 100126791 | EGOT | 3.693632956 | 0.000205704 |
| 84077 | C3orf20 | 3.660466092 | 0.000260804 |
| 116842 | LEAP2 | 3.660466092 | 0.000260804 |
| 128346 | C1orf162 | 3.556129432 | 0.000530144 |
| 3569 | IL6 | 3.532754924 | 2.41E-297 |
| 4314 | MMP3 | 3.519603556 | 5.21E-07 |
| 4929 | NR4A2 | 3.513955612 | 0 |
| 7225 | TRPC6 | 3.482128851 | 0.000848672 |
| 360155 | CYCSP52 | 3.443654703 | 0.00107293 |
| 23237 | ARC | 3.415767745 | 1.59E-66 |
| 338328 | GPIHBP1 | 3.383948457 | 2.66E-06 |
| 27063 | ANKRD1 | 3.372473138 | 2.14E-251 |
| 6445 | SGCG | 3.321664179 | 0.00216058 |
| 100130238 | LOC100130238 | 3.321664179 | 0.00216058 |
| 85235 | HIST1H2AH | 3.300290528 | 6.71E-06 |
| 221584 | ZSCAN12P1 | 3.261001397 | 9.15E-25 |
| 645158 | CBX3P2 | 3.234201338 | 0.00343444 |
| 387978 | C14orf23 | 3.234201338 | 0.00343444 |
| 6364 | CCL20 | 3.223759591 | 9.02E-31 |
| 2209 | FCGR1A | 3.188397648 | 2.11E-05 |
| 3386 | ICAM4 | 3.188397648 | 2.11E-05 |
| 8354 | HIST1H3I | 3.141091933 | 0.00544378 |
| 1844 | DUSP2 | 3.116844387 | 4.18E-05 |
| 51655 | RASD1 | 3.110718284 | 1.69E-17 |
| 8651 | SOCS1 | 3.108670455 | 2.27E-13 |
| 84983 | FAM222A-AS1 | 3.092182333 | 0.0068456 |
| 5743 | PTGS2 | 3.068487114 | 0 |
| 6543 | SLC8A2 | 3.050118273 | 1.10E-12 |
| 50604 | IL20 | 3.04155626 | 8.25E-05 |
| 285782 | CAGE1 | 3.04155626 | 0.00860112 |
| 9586 | CREB5 | 3.023036172 | 2.13E-27 |
| 7634 | ZNF80 | 3.006790841 | 3.35E-12 |
| 1270 | CNTF | 2.98908884 | 0.01079716 |
| 647174 | SERPINE3 | 2.98908884 | 0.01079716 |
| 23764 | MAFF | 2.934641056 | 2.55E-195 |
| 729633 | MRS2P2 | 2.934641056 | 0.000202576 |
| 349149 | GJC3 | 2.934641056 | 0.01354098 |
| 645682 | POU5F1P4 | 2.906626679 | 0.000253208 |
| 79686 | LINC00341 | 2.878057527 | 0.01696486 |
| 81872 | KRTAP2-1 | 2.848911182 | 0.000394872 |
| 5790 | PTPRCAP | 2.848911182 | 0.000394872 |
| 440896 | LOC440896 | 2.788790189 | 0.000614182 |
| 11259 | FILIP1L | 2.770254238 | 1.15E-15 |
| 253559 | CADM2 | 2.749278914 | 0.0212314 |
| 643201 | LOC643201 | 2.693632956 | 1.98E-06 |
| 4050 | LTB | 2.693632956 | 0.001185048 |
| 65072 | CFLAR-AS1 | 2.689770317 | 8.40E-24 |
| 284418 | FAM71E2 | 2.62651876 | 0.001829512 |
| 647288 | CTAGE11P | 2.62651876 | 0.001829512 |
| 23645 | PPP1R15A | 2.621847408 | 0 |
| 9518 | GDF15 | 2.618044215 | 1.80E-229 |
| 5806 | PTX3 | 2.616670974 | 3.36E-61 |
| 2669 | GEM | 2.602074953 | 0 |
| 7128 | TNFAIP3 | 2.575628261 | 0 |
| 8367 | HIST1H4E | 2.570484725 | 5.83E-07 |
| 81796 | SLCO5A1 | 2.556129432 | 0.0028148 |
| 727758 | ROCK1P1 | 2.556129432 | 0.0028148 |
| 346171 | ZFP57 | 2.556129432 | 0.0028148 |
| 84688 | C9orf24 | 2.531881886 | 0.000212008 |
| 29126 | CD274 | 2.526116863 | 9.64E-58 |
| 130540 | ALS2CR12 | 2.519603556 | 0.00348664 |
| 3357 | HTR2B | 2.519603556 | 0.00348664 |
| 2925 | GRPR | 2.519603556 | 0.00348664 |
| 3603 | IL16 | 2.519603556 | 0.00348664 |
| 3624 | INHBA | 2.465098091 | 8.21E-154 |
| 23460 | ABCA6 | 2.443654703 | 0.00533384 |
| 28999 | KLF15 | 2.443654703 | 0.00533384 |
| 7538 | ZFP36 | 2.435616879 | 0 |
| 26784 | SNORA64 | 2.404126339 | 0.00658684 |
| 119548 | PNLIPRP3 | 2.404126339 | 0.00658684 |
| 64109 | CRLF2 | 2.404126339 | 0.00658684 |
| 2049 | EPHB3 | 2.377159291 | 8.92E-07 |
| 1133 | CHRM5 | 2.363484354 | 0.00812512 |
| 407006 | MIR221 | 2.363484354 | 0.00812512 |
| 83715 | ESPN | 2.363484354 | 0.00812512 |
| 7852 | CXCR4 | 2.363484354 | 0.00812512 |
| 124590 | USH1G | 2.363484354 | 0.00812512 |
| 51764 | GNG13 | 2.342725794 | 0.000109108 |
| 142913 | CFL1P1 | 2.278595457 | 0.0002015 |
| 8360 | HIST1H4D | 2.278595457 | 0.01231952 |
| 151306 | GPBAR1 | 2.278595457 | 0.01231952 |
| 100129046 | LOC100129046 | 2.252123246 | 2.63E-09 |
| 8870 | IER3 | 2.248343529 | 0 |
| 10913 | EDAR | 2.234201338 | 0.000302212 |
| 1316 | KLF6 | 2.202609093 | 0 |
| 2921 | CXCL3 | 2.188397648 | 5.25E-58 |
| 51513 | ETV7 | 2.188397648 | 0.01858338 |
| 10716 | TBR1 | 2.188397648 | 0.01858338 |
| 374491 | TPTE2P6 | 2.188397648 | 0.01858338 |
| 100287036 | LOC100287036 | 2.181733918 | 1.20E-11 |
| 283518 | KCNRG | 2.181733918 | 2.31E-06 |
| 146754 | DNAH2 | 2.172800793 | 8.24E-08 |
| 10566 | AKAP3 | 2.172800793 | 0.00313084 |
| 3695 | ITGB7 | 2.153064575 | 6.45E-07 |
| 26768 | RNU105A | 2.141091933 | 0.022776 |
| 65997 | RASL11B | 2.141091933 | 0.022776 |
| 220070 | SHANK2-AS3 | 2.141091933 | 0.022776 |
| 84677 | DSCR8 | 2.141091933 | 0.022776 |
| 375593 | TRIM73 | 2.141091933 | 0.022776 |
| 1848 | DUSP6 | 2.137758789 | 8.58E-183 |
| 2920 | CXCL2 | 2.113285238 | 0.00E+00 |
| 81848 | SPRY4 | 2.105311443 | 1.78E-145 |
| 10888 | GPR83 | 2.092182333 | 1.71E-06 |
| 84941 | HSH2D | 2.075503592 | 0.00569688 |
| 9808 | KIAA0087 | 2.075503592 | 0.00569688 |
| 115362 | GBP5 | 2.058629773 | 1.46E-16 |
| 1839 | HBEGF | 2.053818112 | 2.87E-101 |
| 202299 | C5orf27 | 2.04155626 | 1.31E-15 |
| 81698 | LINC00597 | 2.015561051 | 0.001797622 |
| 8061 | FOSL1 | 2.006671758 | 0 |
| 3589 | IL11 | 2.002051461 | 0 |
| 1649 | DDIT3 | 2.001534786 | 2.91E-229 |
| 7185 | TRAF1 | 1.99384237 | 3.64E-60 |
| 6781 | STC1 | 1.989232728 | 0 |
| 646600 | C3orf65 | 1.98908884 | 0.00218136 |
| 3725 | JUN | 1.982209676 | 0 |
| 64714 | PDIA2 | 1.971166932 | 0.0102562 |
| 51294 | PCDH12 | 1.971166932 | 0.0102562 |
| 650368 | LOC650368 | 1.956667362 | 0.000699278 |
| 6861 | SYT5 | 1.950408372 | 5.12E-05 |
| 1847 | DUSP5 | 1.944290262 | 0 |
| 339166 | LOC339166 | 1.934641056 | 0.0002272 |
| 10253 | SPRY2 | 1.93029465 | 5.52E-97 |
| 337873 | HIST2H2BC | 1.921678749 | 7.90E-115 |
| 4145 | MATK | 1.906626679 | 0.00387306 |
| 57828 | CATSPERG | 1.89716635 | 0.0150786 |
| 9592 | IER2 | 1.883375848 | 0 |
| 728619 | ASB9P1 | 1.878057527 | 0.00467944 |
| 6540 | SLC6A13 | 1.878057527 | 0.00467944 |
| 140876 | FAM65C | 1.858692202 | 0.0182439 |
| 3383 | ICAM1 | 1.85691876 | 2.24E-33 |
| 2827 | GPR3 | 1.856396744 | 4.29E-26 |
| 389170 | LEKR1 | 1.851585316 | 1.59E-06 |
| 59344 | ALOXE3 | 1.844604132 | 9.12E-44 |
| 100507257 | MEG9 | 1.843706023 | 1.48E-17 |
| 128312 | HIST3H2BB | 1.839063396 | 0.000577902 |
| 353322 | ANKRD37 | 1.829147927 | 1.56E-12 |
| 25819 | CCRN4L | 1.825351246 | 1.65E-162 |
| 23135 | KDM6B | 1.824685478 | 0 |
| 3553 | IL1B | 1.819163838 | 0.00680648 |
| 319085 | ITPK1-AS1 | 1.819163838 | 0.0220402 |
| 100506178 | LOC100506178 | 1.819163838 | 0.0220402 |
| 338645 | LUZP2 | 1.819163838 | 0.0220402 |
| 1556 | CYP2B7P1 | 1.819163838 | 0.0220402 |
| 3491 | CYR61 | 1.818168533 | 0 |
| 3781 | KCNN2 | 1.794916292 | 0.00259248 |
| 93082 | NEURL3 | 1.788790189 | 0.0081936 |
| 100507500 | EGFR-AS1 | 1.774769719 | 5.66E-06 |
| 440689 | HIST2H2BF | 1.771776336 | 1.83E-22 |
| 83851 | SYT16 | 1.770254238 | 0.0031134 |
| 100130476 | LOC100130476 | 1.770254238 | 0.0031134 |
| 8336 | HIST1H2AM | 1.757763294 | 7.49E-15 |
| 10256 | CNKSR1 | 1.757763294 | 2.02E-05 |
| 100499489 | LOC100499489 | 1.757763294 | 0.00985054 |
| 151242 | PPP1R1C | 1.743214985 | 1.30E-06 |
| 402176 | RPL21P44 | 1.741995978 | 0.000183824 |
| 6066 | RNU2-1 | 1.740835478 | 3.03E-18 |
| 147744 | TMEM190 | 1.736701678 | 3.87E-06 |
| 283731 | LOC283731 | 1.726054434 | 0.01182656 |
| 51676 | ASB2 | 1.719628165 | 0.0044752 |
| 121504 | HIST4H4 | 1.712248634 | 0.000669068 |
| 346007 | EYS | 1.709934768 | 0.0002624 |
| 3059 | HCLS1 | 1.693632956 | 0.0141791 |
| 338069 | ST7-OT4 | 1.693632956 | 0.0141791 |
| 26238 | C6orf123 | 1.693632956 | 0.0141791 |
| 374407 | DNAJB13 | 1.693632956 | 0.0141791 |
| 100507156 | LOC100507156 | 1.682182935 | 1.99E-27 |
| 1543 | CYP1A1 | 1.681284564 | 1.65E-25 |
| 9074 | CLDN6 | 1.67160665 | 0.00245582 |
| 341208 | HEPHL1 | 1.667160745 | 0.0064024 |
| 399474 | TMEM200B | 1.660466092 | 0.0169749 |
| 8399 | PLA2G10 | 1.660466092 | 0.0169749 |
| 9132 | KCNQ4 | 1.649238837 | 0.0029293 |
| 401024 | FSIP2 | 1.649238837 | 0.0029293 |
| 100505881 | MAGI2-AS3 | 1.646014936 | 2.99E-06 |
| 390 | RND3 | 1.633506821 | 0 |
| 23138 | N4BP3 | 1.629786474 | 1.26E-08 |
| 56477 | CCL28 | 1.62651876 | 0.00349026 |
| 50943 | FOXP3 | 1.62651876 | 0.0202914 |
| 57864 | SLC46A2 | 1.62651876 | 0.0202914 |
| 774 | CACNA1B | 1.619632375 | 3.66E-08 |
| 5801 | PTPRR | 1.618865188 | 0.000294772 |
| 5630 | PRPH | 1.612712961 | 0.0091138 |
| 100128770 | LOC100128770 | 1.609240769 | 0.00074832 |
| 339883 | C3orf35 | 1.606755505 | 5.92E-06 |
| 27 | ABL2 | 1.577410921 | 0 |
| 4084 | MXD1 | 1.576927518 | 4.60E-118 |
| 84070 | FAM186B | 1.562983103 | 6.72E-08 |
| 8365 | HIST1H4H | 1.556129432 | 2.07E-09 |
| 414208 | ZNF32-AS2 | 1.556129432 | 1.37E-05 |
| 644242 | LINC00622 | 1.556129432 | 0.001248738 |
| 4842 | NOS1 | 1.556129432 | 0.00586332 |
| 646329 | LOC646329 | 1.546479262 | 2.32E-24 |
| 84159 | ARID5B | 1.543070397 | 1.87E-276 |
| 91948 | LOC91948 | 1.530903456 | 4.08E-46 |
| 10365 | KLF2 | 1.52727457 | 2.26E-31 |
| 597 | BCL2A1 | 1.526983087 | 3.19E-07 |
| 100130000 | LOC100130000 | 1.526983087 | 0.000381796 |
| 64577 | ALDH8A1 | 1.526983087 | 0.0153233 |
| 1843 | DUSP1 | 1.526876397 | 0 |
| 1942 | EFNA1 | 1.523553284 | 7.30E-188 |
| 10673 | TNFSF13B | 1.519603556 | 0.001748478 |
| 255239 | ANKK1 | 1.519603556 | 0.001748478 |
| 158381 | ATP8B5P | 1.514309257 | 0.00377794 |
| 254778 | C8orf46 | 1.512186085 | 0.00045077 |
| 440288 | LOC440288 | 1.509736527 | 3.36E-24 |
| 9388 | LIPG | 1.507219832 | 0.000118139 |
| 100129858 | LOC100129858 | 1.507219832 | 0.00823578 |
