# Supplementary material for: Transcriptome Profiles of Human Lung Epithelial Cells A549 Interacting with Aspergillus fumigatus by RNA-Seq
Source: PLoS One. 2015 Aug 14;10(8):e0135720. doi: 10.1371/journal.pone.0135720 (PMC4537115; doi:10.1371/journal.pone.0135720)
Supplement: S2 Table — 459 genes were differentially expressed, with p value < 0.05, fold change 1.5 or greater. The fold change for each gene was expressed as the ratio of expression between the two populations, infected A549 cells with A. fumigatus conidia and uninfected A549 cells. Compared with uninfected A549 cells, there were 157 down-regulated genes in A549 cells infected with A. fumigatus conidia. The genes were sorted by fold change. (DOCX) [file pone.0135720.s003.docx]

**Table S2: Down-regulated genes in A549 cells infected with *A. fumigatus* conidia.** 459 genes were differentially expressed, with *p* value < 0.05, fold change 1.5 or greater. The fold change for each gene was expressed as the ratio of expression between the two populations, infected A549 cells with *A. fumigatus* conidia and uninfected A549 cells. Compared with uninfected A549 cells, there were 157 down-regulated genes in A549 cells infected with *A. fumigatus* conidia. The genes were sorted by fold change.

| **GeneID** | **Gene Symbol** | **Fold Change** | **P-value** |
| --- | --- | --- | --- |
| 8363 | HIST1H4J | -8.100454334 | 0.00121188 |
| 797 | CALCB | -7.151321648 | 6.26E-05 |
| 3565 | IL4 | -6.834720778 | 0.00533302 |
| 723972 | ANP32AP1 | -6.633673259 | 0.00533302 |
| 2953 | GSTT2 | -6.426440645 | 0.00121188 |
| 64284 | RAB17 | -6.404832228 | 1.42E-05 |
| 1811 | SLC26A3 | -6.399266653 | 1.67E-07 |
| 83888 | FGFBP2 | -6.361868644 | 0.00121188 |
| 285601 | GPR150 | -6.226353673 | 0.00121188 |
| 7139 | TNNT2 | -6.224144339 | 0.00121188 |
| 1188 | CLCNKB | -6.217536564 | 3.23E-06 |
| 131177 | FAM3D | -6.207681303 | 0.00121188 |
| 100507194 | LOC100507194 | -6.052625809 | 0.00121188 |
| 286187 | PPP1R42 | -6.043507164 | 0.00533302 |
| 100271722 | LOC100271722 | -5.909963762 | 0.00121188 |
| 101180976 | IFNL4 | -5.899349158 | 0.00121188 |
| 93979 | CPA5 | -5.877135921 | 0.000275388 |
| 54754 | NUTMF | -5.838658728 | 6.26E-05 |
| 201617 | LOC201617 | -5.762406573 | 6.26E-05 |
| 139728 | PNCK | -5.693544715 | 0.00121188 |
| 1586 | CYP17A1 | -5.688205632 | 0.00121188 |
| 376412 | RNF126P1 | -5.674315244 | 0.00533302 |
| 6387 | CXCL12 | -5.654346828 | 0.00121188 |
| 283847 | CCDC79 | -5.641907171 | 0.000275388 |
| 100507346 | LOC100507346 | -5.641759724 | 0.00121188 |
| 283483 | LINC00551 | -5.634406522 | 0.00121188 |
| 137835 | TMEM71 | -5.536583247 | 0.00121188 |
| 100652846 | CACNA1C-AS1 | -5.45335977 | 0.00121188 |
| 213 | ALB | -5.431529522 | 0.00121188 |
| 56649 | TMPRSS4 | -5.367953406 | 6.26E-05 |
| 100533990 | APOC4-APOC2 | -5.324310906 | 0.00533302 |
| 633 | BGN | -5.308815833 | 0.00121188 |
| 114569 | MAL2 | -5.109091731 | 0.00121188 |
| 286887 | KRT6C | -4.965778058 | 0.00533302 |
| 64926 | RASAL3 | -4.892315896 | 0.00121188 |
| 8581 | LY6D | -4.765798662 | 2.57E-05 |
| 5349 | FXYD3 | -4.573153585 | 3.75E-08 |
| 2138 | EYA1 | -4.492376057 | 0.00121188 |
| 79656 | BEND5 | -4.350761163 | 0.000401092 |
| 341276 | OR10A2 | -4.350761163 | 0.000401092 |
| 6440 | SFTPC | -4.087726757 | 0.001552242 |
| 100506585 | LOC100506585 | -4.084668664 | 0.00533302 |
| 53405 | CLIC5 | -4.028329259 | 0.00121188 |
| 3205 | HOXA9 | -4.013726176 | 1.08E-09 |
| 80031 | SEMA6D | -3.99946724 | 0.00121188 |
| 728402 | TPI1P3 | -3.765798662 | 0.000112543 |
| 5241 | PGR | -3.765798662 | 0.00589434 |
| 283284 | IGSF22 | -3.765798662 | 0.00589434 |
| 150248 | C22orf15 | -3.765798662 | 0.00589434 |
| 642924 | LINC00535 | -3.573153585 | 0.000407842 |
| 2742 | GLRA2 | -3.573153585 | 0.000407842 |
| 4680 | CEACAM6 | -3.350761163 | 4.90E-08 |
| 80833 | APOL3 | -3.350761163 | 7.97E-06 |
| 3758 | KCNJ1 | -3.350761163 | 0.000105076 |
| 4504 | MT3 | -3.350761163 | 0.001447444 |
| 259236 | TMIE | -3.350761163 | 0.001447444 |
| 285175 | UNC80 | -3.350761163 | 0.001447444 |
| 139599 | MAGEE2 | -3.350761163 | 0.001447444 |
| 203430 | ZCCHC5 | -3.350761163 | 0.0218176 |
| 27006 | FGF22 | -3.350761163 | 0.0218176 |
| 2835 | GPR12 | -3.350761163 | 0.0218176 |
| 3394 | IRF8 | -3.087726757 | 8.97E-05 |
| 85285 | KRTAP4-1 | -3.087726757 | 0.0050057 |
| 303 | ANXA2P1 | -3.087726757 | 0.0050057 |
| 10655 | DMRT2 | -3.070797382 | 0.01674958 |
| 548321 | FAM27A | -2.765798662 | 5.67E-05 |
| 100132103 | FAM66E | -2.765798662 | 0.000227836 |
| 100505483 | PRKAG2-AS1 | -2.765798662 | 0.000929 |
| 404744 | NPSR1-AS1 | -2.765798662 | 0.000929 |
| 692224 | FBXO22-AS1 | -2.765798662 | 0.000929 |
| 54112 | GPR88 | -2.765798662 | 0.00387318 |
| 64067 | NPAS3 | -2.765798662 | 0.01674958 |
| 6886 | TAL1 | -2.765798662 | 0.01674958 |
| 100287879 | LOC100287879 | -2.765798662 | 0.01674958 |
| 142683 | ITLN2 | -2.765798662 | 0.01674958 |
| 165530 | CLEC4F | -2.765798662 | 0.01674958 |
| 644172 | LOC644172 | -2.765798662 | 0.01674958 |
| 7047 | TGM4 | -2.765798662 | 0.01674958 |
| 33 | ACADL | -2.765798662 | 0.01674958 |
| 55784 | MCTP2 | -2.765798662 | 0.01674958 |
| 4322 | MMP13 | -2.765798662 | 0.01674958 |
| 92346 | C1orf105 | -2.765798662 | 0.01674958 |
| 60385 | TSKS | -2.765798662 | 0.01674958 |
| 59272 | ACE2 | -2.765798662 | 0.01674958 |
| 1747 | DLX3 | -2.765798662 | 0.01674958 |
| 2986 | GUCY2F | -2.765798662 | 0.01674958 |
| 100534592 | URGCP-MRPS24 | -2.765798662 | 0.01674958 |
| 27035 | NOX1 | -2.672689258 | 1.09E-05 |
| 63876 | PKNOX2 | -2.573153585 | 0.00286874 |
| 26287 | ANKRD2 | -2.550069971 | 1.06E-09 |
| 79094 | CHAC1 | -2.502764257 | 6.49E-65 |
| 653501 | LOC653501 | -2.502764257 | 0.00051239 |
| 100885782 | MYO16-AS1 | -2.502764257 | 0.0121222 |
| 392197 | USP17L7 | -2.502764257 | 0.0121222 |
| 140902 | R3HDML | -2.443870568 | 0.00207504 |
| 972 | CD74 | -2.350761163 | 0.001480052 |
| 83869 | TTTY14 | -2.350761163 | 0.00855946 |
| 6372 | CXCL6 | -2.350761163 | 0.00855946 |
| 221303 | FAM162B | -2.350761163 | 0.00855946 |
| 154865 | IQUB | -2.350761163 | 0.00855946 |
| 441502 | RPS26P11 | -2.293975137 | 1.23E-32 |
| 388394 | RPRML | -2.25122549 | 0.00597234 |
| 5075 | PAX1 | -2.25122549 | 0.00597234 |
| 55287 | TMEM40 | -2.25122549 | 0.00597234 |
| 171484 | FAM9C | -2.225230281 | 0.00073558 |
| 100131347 | LOC100131347 | -2.180836162 | 8.87E-06 |
| 339778 | C2orf70 | -2.180836162 | 6.68E-05 |
| 199964 | TMEM61 | -2.180836162 | 0.0041415 |
| 388407 | C17orf82 | -2.180836162 | 0.0041415 |
| 100861402 | CERS6-AS1 | -2.152821786 | 4.69E-05 |
| 283385 | MORN3 | -2.05530528 | 0.001361422 |
| 4902 | NRTN | -2.013726176 | 4.07E-05 |
| 100271874 | ZNRF2P2 | -1.988191084 | 0.0111687 |
| 80099 | C7orf69 | -1.988191084 | 0.0111687 |
| 94027 | CGB7 | -1.95844374 | 6.58E-06 |
| 4958 | OMD | -1.95844374 | 0.00756336 |
| 346689 | KLRG2 | -1.917801756 | 0.00348968 |
| 56136 | PCDHA13 | -1.903302186 | 0.00237704 |
| 4188 | MDFI | -1.891329545 | 0.001621844 |
| 3898 | LAD1 | -1.891329545 | 0.001621844 |
| 780851 | SNORD3A | -1.886092896 | 8.05E-19 |
| 11223 | MST1L | -1.858908067 | 0.000356392 |
| 100507250 | LOC100507250 | -1.848260823 | 0.000168197 |
| 100133286 | LOC100133286 | -1.816424736 | 2.85E-06 |
| 100129726 | LOC100129726 | -1.765798662 | 1.67E-07 |
| 26047 | CNTNAP2 | -1.765798662 | 0.000579714 |
| 100302739 | PCNA-AS1 | -1.765798662 | 0.00267844 |
| 92745 | SLC38A5 | -1.765798662 | 0.00267844 |
| 64396 | GMCL1P1 | -1.765798662 | 0.00583076 |
| 1805 | DPT | -1.765798662 | 0.00864078 |
| 6414 | SEPP1 | -1.765798662 | 0.0128519 |
| 121340 | SP7 | -1.765798662 | 0.0128519 |
| 645528 | LINC00264 | -1.765798662 | 0.0128519 |
| 100861437 | NARR | -1.765798662 | 0.0128519 |
| 407977 | TNFSF12-TNFSF13 | -1.765798662 | 0.0128519 |
| 3101 | HK3 | -1.765798662 | 0.0191998 |
| 816 | CAMK2B | -1.691798081 | 0.000293968 |
| 93659 | CGB5 | -1.672689258 | 0.001339148 |
| 2706 | GJB2 | -1.666262989 | 0.001963832 |
| 1048 | CEACAM5 | -1.656864291 | 8.21E-09 |
| 9627 | SNCAIP | -1.65476735 | 4.80E-05 |
| 1080 | CFTR | -1.64026778 | 0.00627154 |
| 645683 | RPL13AP3 | -1.628295139 | 0.00928448 |
| 100500862 | MIR3648 | -1.613795569 | 0.0137919 |
| 4168 | MCF2 | -1.613795569 | 0.0137919 |
| 255762 | PDZD9 | -1.595873661 | 0.0205714 |
| 4815 | NINJ2 | -1.564164801 | 0.000466824 |
| 150786 | WTH3DI | -1.559347785 | 0.00441952 |
| 154796 | AMOT | -1.543406241 | 0.000988032 |
| 150084 | IGSF5 | -1.531333409 | 0.00143955 |
| 8788 | DLK1 | -1.517871149 | 1.78E-05 |
| 388564 | TMEM238 | -1.502764257 | 4.95E-07 |
| 2902 | GRIN1 | -1.502764257 | 0.01417746 |
| 121601 | ANO4 | -1.502764257 | 0.01417746 |
| 652995 | UCA1 | -1.502764257 | 0.01417746 |
| 93664 | CADPS2 | -1.502764257 | 0.01417746 |
| 1117 | CHI3L2 | -1.502764257 | 0.01417746 |
